# Notes on the genus *Tunga* (Siphonaptera: Tungidae) II – neosomes, morphology, classification, and other taxonomic notes

**DOI:** 10.1051/parasite/2014067

**Published:** 2014-12-17

**Authors:** Pedro Marcos Linardi, Jean-Claude Beaucournu, Daniel Moreira de Avelar, Sorya Belaz

**Affiliations:** 1 Departamento de Parasitologia, Instituto de Ciências Biológicas, Universidade Federal de Minas Gerais, Caixa Postal 486, Avenida Presidente Antônio Carlos, 6627, Campus UFMG Belo Horizonte Minas Gerais 31270-901 Brazil; 2 Laboratoire de Parasitologie et Zoologie appliquée, Faculté de Médecine 2, avenue du Professeur Léon Bernard 35043 Rennes Cedex France; 3 Institut de Parasitologie de l’Ouest, Faculté de Médecine 2, avenue du Professeur Léon Bernard 35043 Rennes Cedex France; 4 Laboratório de Pesquisas Clínicas, Centro de Pesquisas René Rachou, Fundação Oswaldo Cruz Belo Horizonte Minas Gerais Brazil; 5 Laboratoire de Parasitologie, Mycologie et Immunologie parasitaire, Centre Hospitalier Régional Universitaire 2 rue Henri Le Guilloux 32033 Rennes Cedex France

**Keywords:** *Tunga*, Siphonaptera, Sand fleas, Neosomes, Morphology, Taxonomy

## Abstract

This review focuses on the neosomes, morphology, and taxonomy of adult species of the genus *Tunga*, complementing the previously published data on the phylogeny, ecology, and pathogenic role. Neosomes are structures formed after penetration of adult females into the skin of hosts resulting in significant enlargement, being the most characteristic and most frequently observed form in hosts. Neosomes can be differentiated by shape, measurements, and sites of attachment to principal hosts. The taxonomic value and morphometric data of the most widely used characteristics to separate species – such as frontal curvature, head chaetotaxy, preoral internal sclerotization, ventral and dorsal genal lobes, eyes, maxillary palps, fusion of pronotum and mesonotum, metacoxae, metatarsi chaetotaxy, spermatheca (females), manubrium, basimere, telomere, and phallosome (males) – are comparatively analyzed. The sexes, individual variations, undescribed species, higher taxa, as well as a proposal for division of the genus into two subgenera (*Tunga* and *Brevidigita*) are presented (as previously given by Wang). A key for females, males, and gravid females (neosomes) also is included for identifying the 13 known species. Data on host specificity and geographical distribution may also support the identification of *Tunga* species because some sand fleas and their hosts may have co-evolved.

## Abstract (fr)

Ce travail est axé sur les néosomes, la morphologie et la taxonomie des adultes du genre *Tunga,* complétant les données déjà publiées sur la phylogénie, l’écologie et le rôle pathogène de ces espèces. Les néosomes sont des structures élaborées après la pénétration de la femelle adulte dans les téguments de l’hôte: ils montrent une taille significativement plus grande et sont les formations les plus caractéristiques et les plus fréquemment rencontrées chez les hôtes. Les néosomes peuvent être différenciés par leur forme, leurs dimensions et les zones de localisation sur leurs principaux hôtes. La valeur taxonomique et les données morphométriques des caractères les plus utilisés pour la séparation des espèces – tels que la courbure frontale, la chétotaxie céphalique, les sclérifications préorales internes, le lobe génal interne et externe, la forme et le développement de l’œil, la conformation des palpes maxillaires, le degré de fusion du pronotum et du mesonotum, la forme et la chétotaxie des metacoxae, la chétotaxie des métatarses, chez les femelles la forme de la spermathèque, chez les mâles, la forme du manubrium, du basimère, du télomère et du phallosome, sont comparées et discutées. Les sexes, les variations individuelles, les taxa non décrits, les classifications de plus haut niveau, de même qu’une proposition (déjà donnée par Wang) de diviser ce genre en deux sous-genres (*Tunga* and *Brevidigita*), sont exposés. Une clé des 13 espèces décrites est proposée pour les femelles, les mâles et les femelles gravides, ou néosomes. La spécificité et la distribution géographique peuvent également aider à l’identification des espèces de *Tunga* car diverses puces-chiques semblent avoir co-évolué avec leurs hôtes.

## Introduction

Currently, the genus *Tunga* comprises 13 species, representing less than 0.5% of the world flea fauna, which consists of approximately 3,000 known species [27]. One species, *T. penetrans* (L., 1758) [35], presents a wide distribution and a high degree of specificity occurring in the Neotropical region and sub-Saharan Africa, in spite of occasional reports of individuals from the USA [7, 45], Europe [49], and New Zealand [42] in whom sand flea infestations were diagnosed after travel to infested regions. Two species, *T. caecigena* Jordan and Rothschild, 1921 [24] and *T. callida* Li and Chin, 1957 [29], parasitize essentially commensal rats in the Oriental Region, while *T. monositus* Barnes and Radovsky, 1969 [2] infests wild rodents in the southwestern USA [19]. Nine other species occur in the Neotropics; four are found on wild and commensal rodents [6, 20, 37]: *T. caecata* (Enderlein, 1901) [15]; *T. libis* Smit, 1962 [46]; *T. bossii* De Avelar, Linhares, and Linardi, 2012 [13] and *T. bonneti* Beaucournu and González-Acuña, 2012 [5, 6]; three infest edentates [21, 30, 34]: *T. travassosi* Pinto and Dreyfus, 1927 [43]; *T. bondari* Wagner, 1932 [50] and *T. terasma* Jordan, 1937 [22]; and two occur on domestic Artiodactyla and man [38, 39, 41]: *T. trimamillata* Pampiglione, Trentini, Fioravanti, Onori, and Rivasi, 2002 [40]; and *T. hexalobulata* De Avelar, Facury Filho and Linardi, 2013 [11]. It is important to stress that more than 30% of the species have been described since 2002, and 23% of these species were described only in the last 2 years, indicating that opportunities for new findings are abundant.

Among the Siphonaptera, *Tunga* is the most specialized genus because the adult females penetrate into the skin of their hosts. Similar to other fleas, both males and females are blood-feeding [18, 53], however, the larvae and adult male of *T. monositus* do not feed [26].

Recently, a review of the genus *Tunga* concerning taxonomy, phylogeny, ecology and pathogenic role was presented by Beaucournu et al. [5], although the review does not include *T. hexalobulata*, which was described subsequently. Similarly, De Avelar et al. [13], when describing *T. bossii* for the first time, presented a widely used dichotomous key for identifying the known species and their neosomes and excluded *T. bonnetti* and *T. hexalobulata,* which were discovered later.

The present study complements this review with regard to the neosomes, morphology, classification, and other taxonomic notes. The taxonomic value and morphometric data of several characteristics are comparatively analyzed. Although the morphological aspects are often not known for many taxa, here we present a new key for identifying the 13 known *Tunga* species, including a more classical vision and emphasizing neosomal characteristics.

## Neosomes

Neosomes are altered organisms resulting from a process characterized by the growth of new tissue and the formation of a new morphological structure accompanied by significant enlargement after adult eclosion [1]. This phenomenon, previously designated by Jordan [23] as teleomorphosis, is known as neosomy and occurs among several arthropods, including queen ants (Formicidae), queen termites (Termitidae), termitophilus beetles (Staphylinidae), Diptera (Phoridae, Streblidae, Carnidae), parasitic Copepoda, Acari (Trombiculidae, Ixodidae [but not Argasidae], Pyemotidae), and some Siphonaptera.

Approximately 90 species of fleas have sessile or semi-sessile females that, after eclosion, remain more or less permanently attached to the integument of the host [44]. Considering the permanence of the hypertrophied females on the hosts, these fleas can be classified as (i) internal, in the host beneath the skin (mesoparasites): *Tunga* (Tungidae) and *Neotunga* (Pulicidae); (ii) an external, sessile female, permanently attached as soon as it is on its host (ectoparasites): *Echidnophaga* (Pulicidae), *Hectopsylla* (Tungidae); or (iii) an external, non-sessile female, temporarily attached when on its host: *Chaetopsylla pro parte*, *Dorcadia, Vermipsylla* (Vermipsyllidae), *Parapsyllus pro parte* (Rhopalopsyllidae), *Glaciopsyllus* (Ceratophyllidae), *Ancistropsylla* (Ancistropsyllidae), *Malacopsylla* and *Phthiropsylla* (Malacopsyllidae).

**Figure 1.**
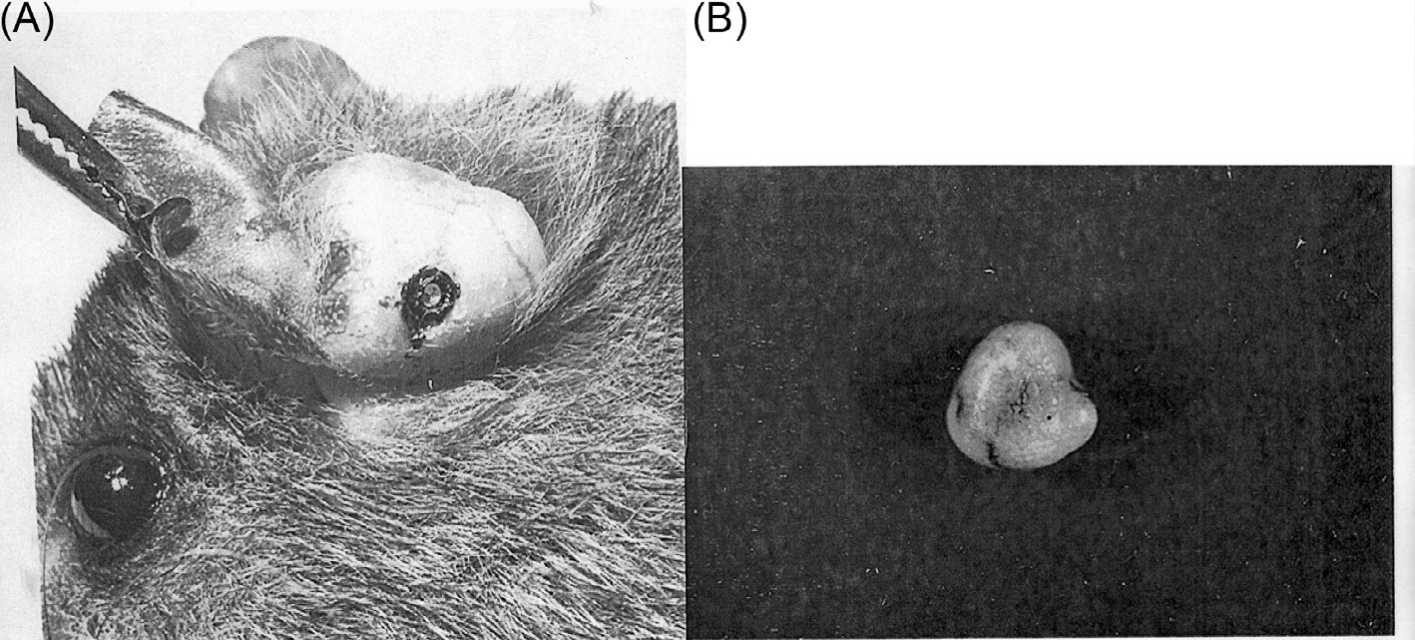
A neosome of the *caecata* group of species: (A) embedded on the ear of *Nectomys squamipes* (×3); (B) frontal view after extraction (×12).

**Figure 2.**
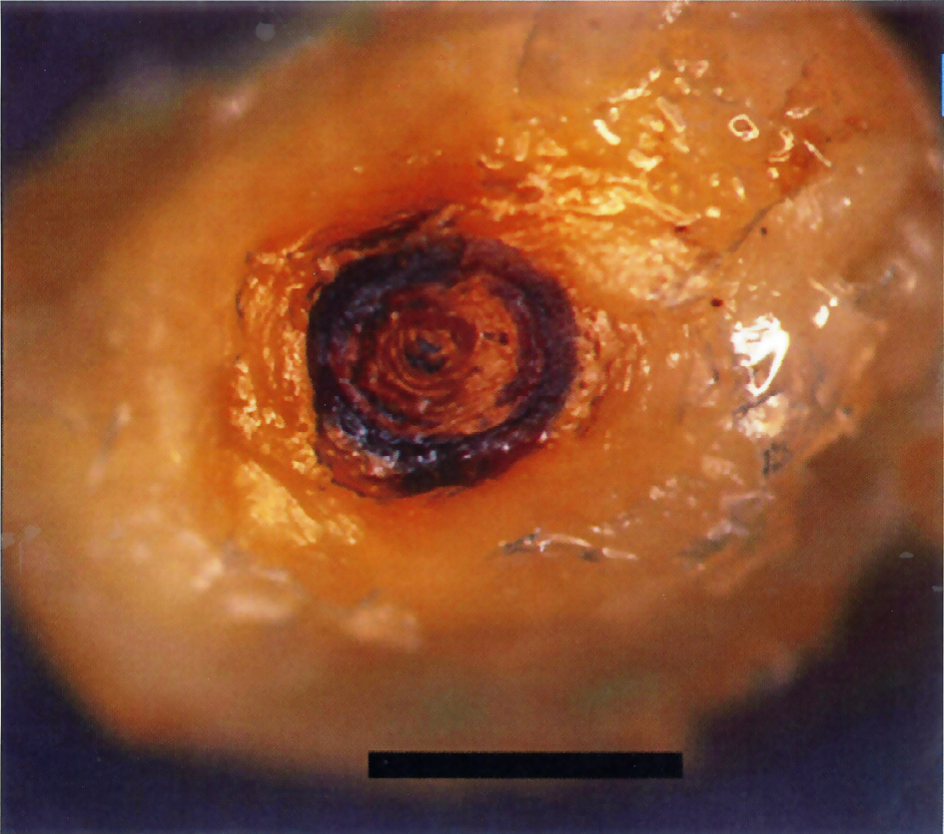
A neosome of *Tunga penetrans* – posterior view. Scale bar = 2 mm.

Morphological studies of the genus *Tunga* have concentrated on the description of the neosomes because they are the most characteristic and most frequently observed form in hosts. Females and males measure approximately 1 mm, but after penetration, the gravid females increase considerably in size, reaching approximately 10 mm (Fig. 1), and expose, on the surface of hosts, only the vital respiratory, anal, and vaginal apertures, which are contained in a caudal disk or conical prominence. The caudal disk exhibits some differences among species: (i) flattened, as in *T. penetrans* (Fig. 2), *T. monositus*, and *T. bonneti*; (ii) conical, as in *T. caecata*, *T. travassosi*, *T. trimamillata*, and *T. hexalobulata*; or (iii) cylindrical, as in *T. terasma*, *T. bondari*, *T. caecigena*, and *T. callida*. Otherwise, the caudal disk can be (i) as wide as long, or almost as wide as long, as in *T. caecata*, *T. travassosi*, and *T. callida*; (ii) wider than long, as in *T. penetrans*, *T. monositus*, *T. trimamillata*, *T. bossii*, and *T. hexalobulata*; or (iii) longer than wide, as in *T. terasma*, *T. bondari*, and *T. caecigena*. Neosomes can live more than three months attaching to different sites of their respective hosts [14, 26]. Consequently, neosomes are important for identifying the species in the genus *Tunga*.

A review solely on the neosomes of tungid fleas that infest wild and domestic animals and concentrated mainly on hosts, infestation, sites of attachment, and impact on the hosts was recently presented by Linardi and Avelar [32].

Table 1 shows the known *Tunga* species with their respective geographic distributions, sexes, stages, neosomes, and sites of attachment to principal hosts. Excepting neosomes, in which the size is indicated in millimeters, all measurements included in other tables are in micrometers.

**Table 1.** *Tunga* species: Geographic distribution, sexes, stages, neosomes, and sites of attachment to their principal true hosts.

*Tunga* species (Geographical distribution)	Sexes and stages	Neosome (hypertrophied female)		
			Measurements (mm)	Main true hosts
		Shape	(length × width × height)	**Order**: *Genera* (principal sites of attachment)
*T. penetrans* (Latin America, Africa)	♂, ♀, hypertrophied ♀, egg, larva 1st instar, larva 2nd instar, pupa	Globular without lobes	6 × 5 × 4	**Artiodactyla**: *Sus* (feet, scrotum, snout),
				**Primates**: *Homo* (between the toes, periungueal region)
				**Carnivora**: *Canis* (around the claws, on the pads, on the muzzle)
				**Cingulata**: *Dasypus* (feet and paws)
				**Rodentia**: *Rattus*, *Mus* (feet)
*T. caecata* (Brazil)	♀, hypertrophied ♀	Globular without lobes	7 × 6 × 6	**Rodentia**: *Rattus*, *Mus* (upper surface of ears)
*T. travassosi* (Brazil)	hypertrophied ♀	Globular without lobes	13 × 8 × 10	**Cingulata**: *Dasypus* (ventral abdominal region)
*T. terasma* (Brazil)	♂, ♀ (?), hypertrophied ♀	With four prominent subcylindrical lobes	10 × 9 × 13	**Cingulata**: *Euphractus, Dasypus, Cabassous, Priodontes* (ventral abdomen and toes)
*T. bondari* (Brazil)	hypertrophied ♀	Mushroom-shaped with a stem and conical posterior region	6 × 6 × 5	**Pilosa**: *Tamandua* (ventral abdomen)
*T. caecigena* (China, Japan)	♂, ♀, hypertrophied ♀	Elliptical with four lobes: dorsal and ventral portions of similar dilatation	7–10 × 5 × 6	**Rodentia**: *Rattus, Mus* (edge of the pinna and dorsal surface of ears)
*T. callida* (China)	♂, ♀, hypertrophied ♀	Spherical with four lobes: dorsal portion more swelled than the ventral portion	4.5 × 4.5 × 4.5	**Rodentia**: *Rattus, Mus* (around the anus)
*T. libis* (Ecuador, Chile)	♂, ♀, hypertrophied ♀	Vertically elliptical and without lobes	Higher than long	**Rodentia**: *Akodon, Phyllotis* (ears)
*T. monositus* (USA, Mexico)	♂, ♀, hypertrophied ♀, egg, larva 1st instar, larva 2nd instar, pupa	Bell-shaped with eight lobes, arranged as four large outer lobes and four small inner lobes	6 × 5.4 × 4.5	**Rodentia**: *Peromyscus, Neotoma* (upper surface of the pinna)
*T. trimamillata* (Ecuador, Peru, Brazil)	♂, ♀, hypertrophied ♀	Globular with three lobes located anteriorly	12 × 5 × 5	**Artiodactyla**: *Bos* (coronary band, sole of the hoof, perianal area, udder, prepuce) **Primates**: *Homo* (feet)
*T. bossii* (Brazil)	hypertrophied ♀	Globular without lobes	9 × 8 × 7	**Rodentia**: *Delomys* (base of the tail)
*T. bonneti* (Chile)	♂, ♀, hypertrophied ♀	Horizontally elliptical without lobes, in form of rugby ball	10 × 6 × 6	**Rodentia**: *Phyllotis* great axis of the tail)
*T. hexalobulata* (Brazil)	hypertrophied ♀	Spherical, with six lobes located anteriorly, pearl-white colored, slightly compressed in anterior direction	4 × 4 × 4	**Artiodactyla**: *Bos* (coronary band)

## Morphology

Excepting neosomes, the classical structures more frequently used to separate or group the species of *Tunga* are discussed as follows:

### Head (Fig. 3)

**Figure 3.**
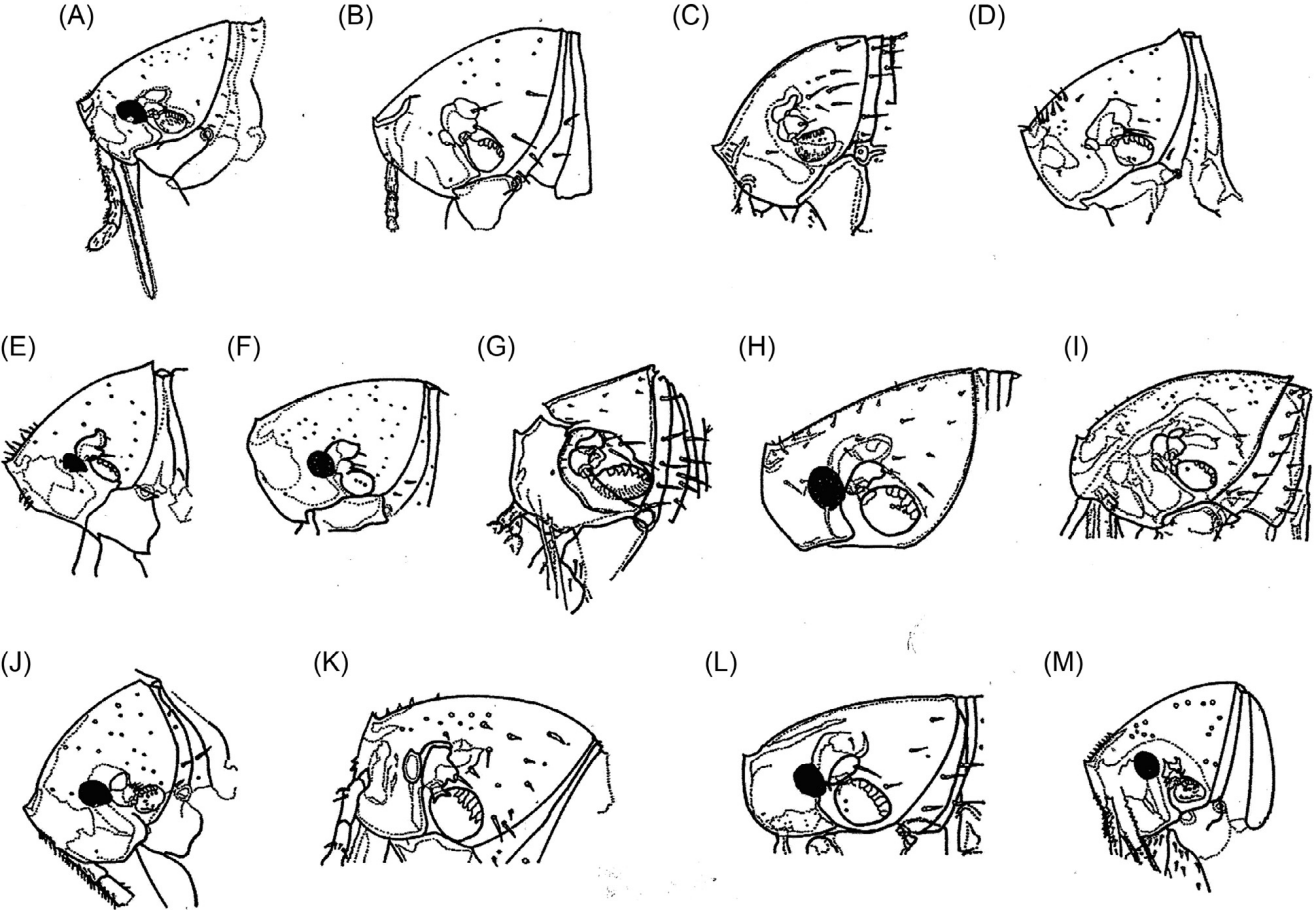
The heads of some species of *Tunga*: (A) *T. penetrans*; (B) *T. caecata*; (C) *T. caecigena*; (D) *T. travassosi*; (E) *T. bondari*; (F) *T. terasma*; (G) *T. callida*; (H) *T. libis*; (I) *T. monositus*; (J) *T. trimamillata*; (K) *T. bossii*; (L) *T. bonneti*; (M) *T. hexalobulata*.

The *cephalic capsule* includes the majority of the characteristics used for identifying these species. Members of the genera *Tunga*, *Hectopsylla* (with the exception of the female of *Hectopsylla coniger*), *Echidnophaga*, *Phacopsylla*, and *Neotunga euloidea* are always characterized by an angular profile and a well-pronounced frontal tubercle, as in *T. caecigena* (Fig. 3C), *T. travassosi* (Fig. 3D), *T. bondari* (Fig. 3E), *T. trimamillata* (Fig. 3J), *T. bossii* (Fig. 3K), and *T. hexalobulata* (Fig. 3M). The *front curvature* varies little but is gently convex or sharper as in *T. caecigena* (Fig. 3C) or almost straight as in *T. libis* (Fig. 3H). Generally, the ventral profile of the cephalic capsule shows a *genal lobe*, which is often more pronounced in females; it seems absent in *bossii*, but is very clear in *caecata*, *caecigena*, *travassosi*, *bondari*, *terasma*, and *monositus*. *Preoral internal sclerotization* presents as the posterior arm longer than the anterior arm (*caecata*, *monositus*, *trimamillata*, and *hexalobulata*) or with the posterior arm subequal to or shorter than the anterior arm (*caecigena*, *penetrans*, *bondari*, *bossii*), or much shorter than the anterior arm (*callida*). A *dorsal genal lobe* either covers part of the antennal segment III (*bondari*, *travassosi*, *caecigena*, *callida*) or not, as in other species. The number of bristles on the postantennal region, posterior region to the frontal tubercle, antennal segment II, and base of the maxilla may be a differential characteristic. Inside the cephalic capsule, the *eyes* are the most characteristic structures for species identification. They may be absent in *caecigena* and *callida* or present in other species, or they may be pigmented (*penetrans*, *bondari*, *terasma*, *libis*, *trimamillata*, *bonneti*, and *hexalobulata*) or without black pigment (*caecata*, *travassosi*, *monositus*, and *bossii*). When present, the eyes can be small (*caecata*, *travassosi*, and *monositus*), or large, in the case of other species. In some species such as *penetrans*, *bondari*, *trimamillata,* the eyes also have a recess into their internal margins. The greatest eye diameters and the numeric variations of bristles on some structures of *Tunga* species are presented in Table 2.

**Table 2.** Eye and numeric variations of bristles on some structures of *Tunga* species.

Species of *Tunga*	Eye: pigmentation/measurements (µm)/presence or absence of recess into its internal margin	Number of bristles on the			
		Postantennal region	Posterior region to the frontal tubercle	Antennal segment II	Base of the maxilla
*T. penetrans*	Pigmented, 61.2 × 44.3, without recess	12–14	8	4	6
*T. caecata*	Unpigmented, 24.6 × 17.2, without recess	12–15	8	1	1
*T. travassosi*	Unpigmented, 35.1 × 27.1, with recess	10	9	2	1
*T. terasma*	Pigmented, 62.5 × 49.2, with recess	20	6	2	3
*T. bondari*	Pigmented, 52.5 × 27.1, with recess	10	8	1	3
*T. caecigena*	Absent	8–10		1	1
*T. callida*	Absent	3–5	6	2 (?)	
*T. libis*	Pigmented, without recess	12	8	1	1
*T. monositus*	Unpigmented, without recess, narrow and inconspicuous	10	8	1	0
*T. trimamillata*	Pigmented, 59.0 × 46.8, with recess	17–19	8	4	6–7
*T. bossii*	Unpigmented, 36.9 × 22.1, without recess	10	6	1	2
*T. bonneti*	Pigmented, with recess			1	
*T. hexalobulata*	Pigmented, 63.4 × 55.3, without recess	9	8	3	6

**Figure 4.**
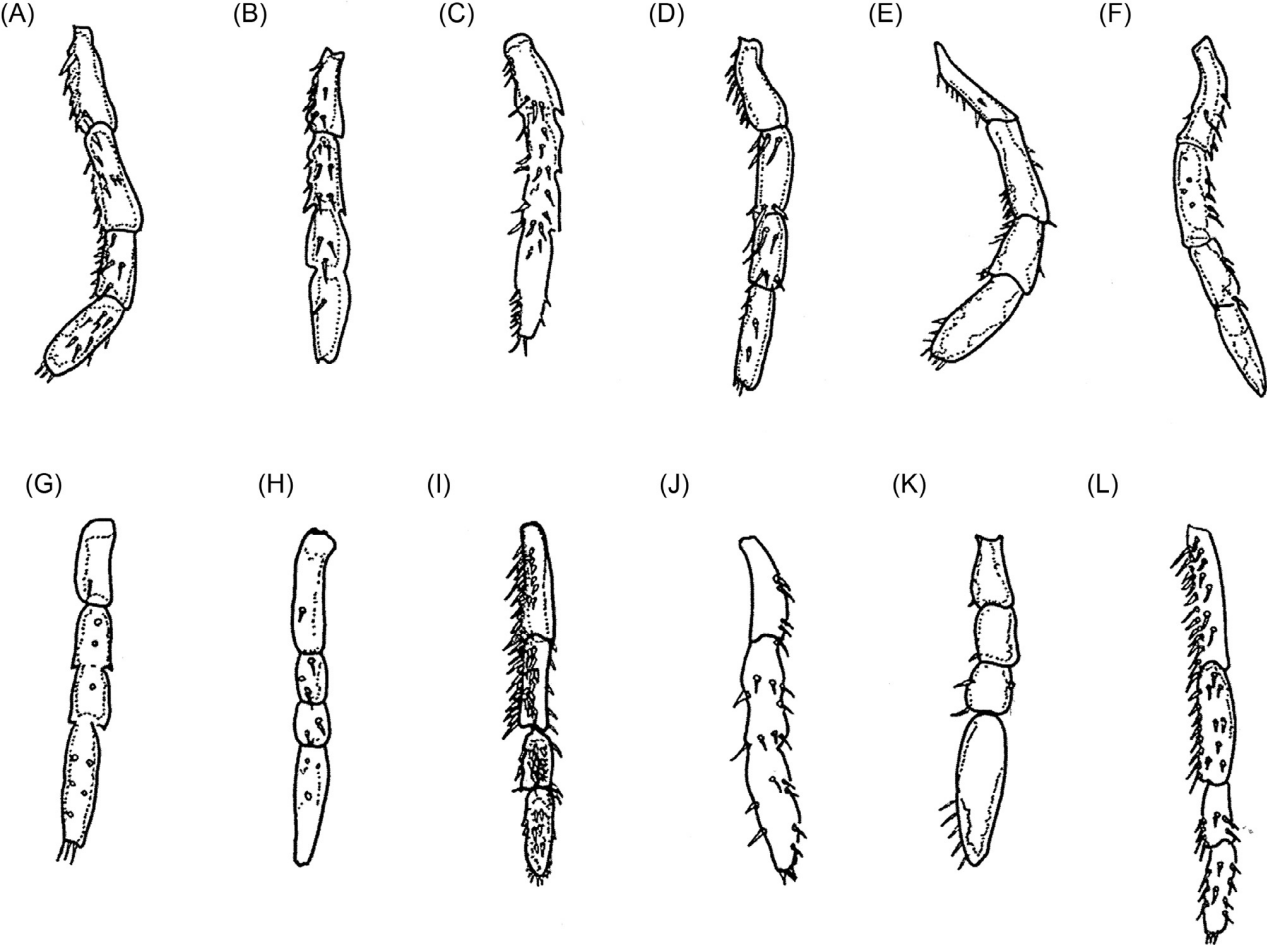
The maxillary palps of some species of *Tunga*: (A) *T. penetrans*; (B) *T. caecata*; (C) *T. caecigena*; (D) *T. travassosi*; (E) *T. bondari*; (F) *T. terasma*; (G) *T. callida*; (H) *T. monositus*; (I) *T. trimamillata*; (J) *T. bossii*; (K) *T. bonneti*; (L) *T. hexalobulata*.

Maxillary palps (Fig. 4) with segments vary in size and chaetotaxy; in *caecigena*, *callida*, and *bonneti*, segment IV is longer than segment I, though the reverse is true in *T. trimamillata* and *T. hexalobulata*. In other species, the palps are approximately the same length. In *T. bossii*, segment I was described as very small and segments III and IV as being incompletely divided; however, a new observation showed that, in fact, what was thought to be “segment I” was a strengthened area at the base of the palp and an incomplete division occurred between segments II and III and between segments III and IV.

Morphometric data regarding the maxillary palps, including new measurements for *T. bossii*, laciniae and preoral internal sclerotization are indicated in Table 3.

**Table 3.** Morphometric variations in maxillary palps and laciniae in *Tunga* species.

Species of *Tunga*	Maxillary palps (MP)		Laciniae (Lac)		Preoral internal sclerotization
	Measurements (µm)	In decreasing order of length	Measurements (µm)	Ratio Lac/MP	Ratio posterior arm/anterior arm
*T. penetrans*	I = 71.2; II = 75.0; III = 52.5; IV = 67.5	II = I = IV > III; II = 1.5 > III	362.5	1.36	1.53
*T. caecata*	I = 45.0; II = 40.0; III = 30.0; IV = 52.5	IV > I = II > III	257.5	1.53	10.00
*T. travassosi*	I = 69.5; II = 63.9; III = 53.5; IV = 84.9	IV > I > II > III; IV = 1.5 > III	336.4	1.23	2.00
*T. terasma*	I = 85.0; II = 82.5; III = 60.0; IV = 77.5	I = II = IV > III; III = 2/3 II	400.0	1.31	1.50
*T. bondari*	I = 55.0; II = 70.0; III = 37.5; IV = 62.5	II > IV > I > III; II = 2 > III	455.0	2.02	2.00
*T. caecigena*		IV > I = II > III		~1.0	3.00
*T. callida*		IV > I > II > III*		1.5	3.00
*T. libis*		IV > I = II > III; IV = 2 > III		1.5	1.50
*T. monositus*	I = 78.0; II = 40.0; III = 35.0; IV = 81.0	I = IV > II ≥ III	330.0	1.4	2.00
*T. trimamillata*	I = 110.7; II = 73.8; III = 44.3; IV = 68.9	I > II = IV > III; I = 2 > III	403.4	1.35	3.98
*T. bossii*	I = 49.2; II = 31.5; III = 30.0; IV = 71.3	IV > I > II > III	233.7	1.28	2.00
*T. bonneti*		IV > I = II = III		1.5	
*T. hexalobulata*	I = 122.5; II = 92.0; III = 52.0; IV = 74.3	I > II > IV > III; I = 2 > III	434.0	1.27	4.68

* Calculated from the figures included in Liu et al. [36]: *Fauna sinica*, p. 139, Figure 54.

### Thorax

**Figure 5.**
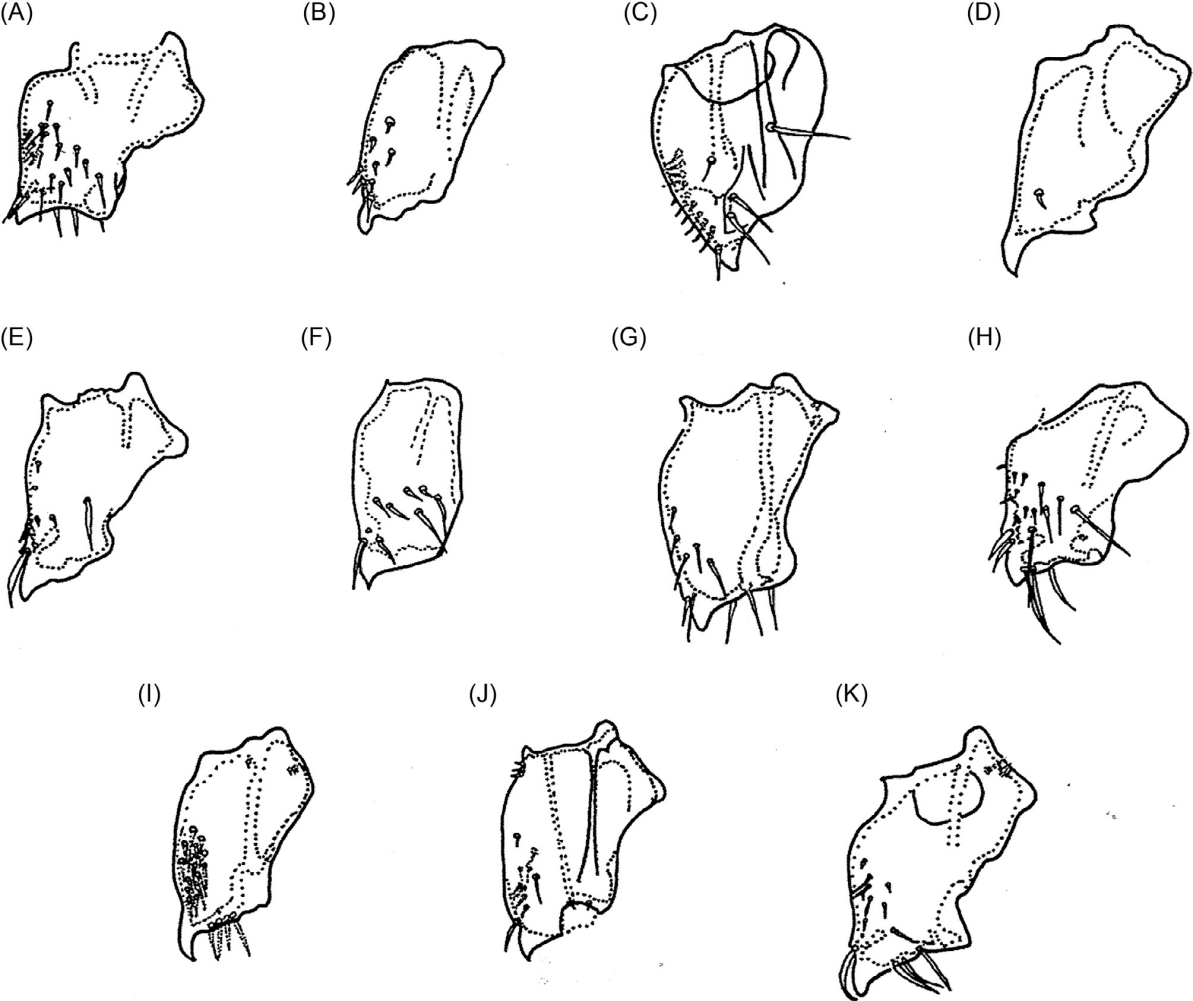
The metacoxae of some species of *Tunga*: (A) *T. penetrans*; (B) *T. caecata*; (C) *T. caecigena*; (D) *T. travassosi*; (E) *T. bondari*; (F) *T. terasma*; (G) *T. monositus*; (H) *T. trimamillata*; (I) *T. bossii*; (J) *T. bonneti*; (K) *T. hexalobulata*.

**Figure 6.**
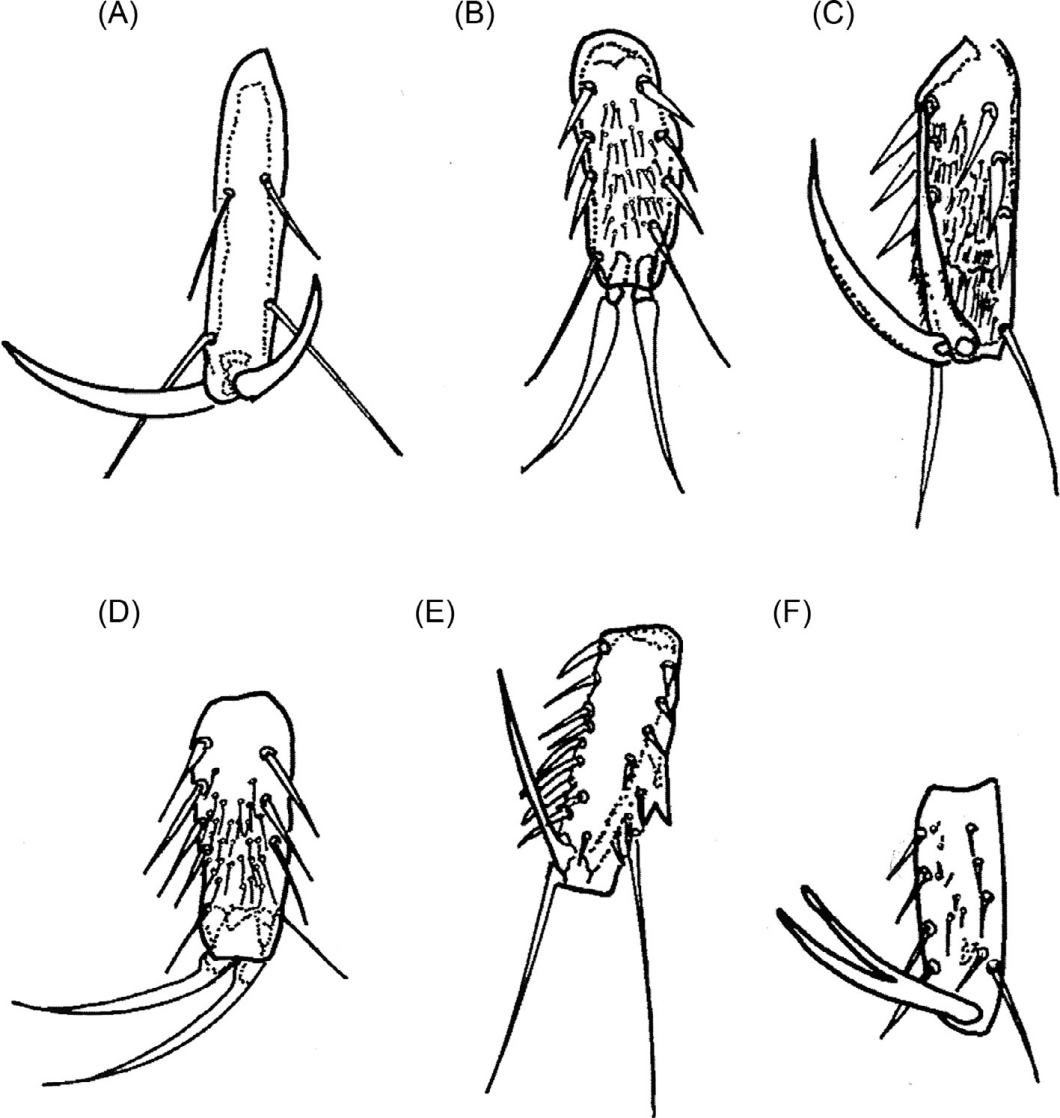
The metatarsi of some species of *Tunga*: (A) *T. penetrans*; (B) *T. caecigena*; (C) *T. callida*; (D) *T. libis*; (E) *T. monositus*; (F) *T. bonneti*.

As Smit [46] noted, the *fusion of the pronotum and mesonotum* can be dorsally complete, a characteristic of the *penetrans* group or incomplete, as in the *caecata* group. Currently, the *penetrans* group includes *T. penetrans*, *T. travassosi*, *T. bondari*, *T. terasma*, *T. trimamillata*, and *T. hexalobulata* [11, 13]. In the *caecata* group, the following species are included: *T. caecata*, *T. caecigena*, *T. callida*, *T. libis*, *T. monositus*, *T. bossii*, and *T. bonneti*. The *chaetotaxy* is generally sparse, consisting of 1–7 bristles on the prothorax. *Legs III* show the classical increase in length, compared to the first two pairs, typical of Siphonaptera. However, these legs are slender, and members of the *Tunga* genus are poor jumpers despite the existence of a pleural arch. At this leg, Beaucournu et al. [5] noted an external guard tooth, whose size is variable depending on the species and ranges from completely absent to normally developed. The *coxae* are always preserved regardless of the autotomy of the legs. The *metacoxae* (Fig. 5) project downward at an anterodistal angle, like a wide tooth, and exhibit a variable number of bristles both on the external and internal surfaces. Species such as *penetrans*, *trimamillata*, and *hexalobulata* present a greater number of bristles. In the species of the *caecata* group, the *metacoxae* are slightly wider at the base than at the apex (Figs. 5E, H, I), though in the *penetrans* group, excepting *T. terasma* (Fig. 5F), the basal part is nearly two times wider than the apical part (Figs. 5A, B, H, J, K). According to Hopkins and Rothschild [20], *T. penetrans* is characterized by a slower and less complete deterioration of the legs than the other species known at that time. The chaetotaxy of *tibiae, tarsi*, and especially of the distal segment of leg III are discriminant characteristics. According to Smit [46], the chaetotaxy of the distal tarsal in the species belonging to the *penetrans* group is strongly reduced, with only two pairs of hair-like lateral plantar bristles and no patch of minute plantar setae segment bristles (Fig. 6A). In contrast, the chaetotaxy is only slightly reduced in the species of the *caecata* group, which exhibit three or four pairs of stiff subspiniform lateral plantar bristles and a patch of minute plantar bristles (Figs. 6B–F). Unfortunately, these articles are often missing even when studying a female that is recently embedded.

### Abdomen

Externally, the most striking distinguishing feature between the *penetrans* and *caecata* groups is the variation in the size of the spiracles of the hypertrophied females. In the *penetrans* group, the spiracles of terga II–IV have disappeared, while in the *caecata* group, they are smaller than the others, though present [46]. The *spermatheca* presents a certain enigma. In the female neonate or a female not yet embedded, this structure is invisible upon first examination, as far as we know for the fleas that have a body or *bulga*, is most often sclerotized, and is a distal appendage, tail, or *hilla* that acts as a pump for sperm. In fleas that are not attached, a thorough examination will reveal, however, a transparent area called *cribiform area*, which is riddled with multiple orifices, at the base of the bulga. As in other genera, it is connected to the vagina through a duct, the *ductus spermathecae*, a visible though poorly defined structure. The spermatheca itself is diaphanous, and more or less conical in some species such as *penetrans* or *trimamillata.* In contrast, this structure is heavily sclerotized in neosomatic and fertilized females and has a consistent shape within a given species. Both Karsten [25] and Bonnet [8] described the spermatheca perfectly in the *penetrans group*, although Bonnet was not able to identify its purpose. Bonnet also noted the presence of an occasional double spermatheca in *T. penetrans*, which was omitted by Beaucournu et al. [4] and is, thus far, unique to this family.

**Figure 7.**
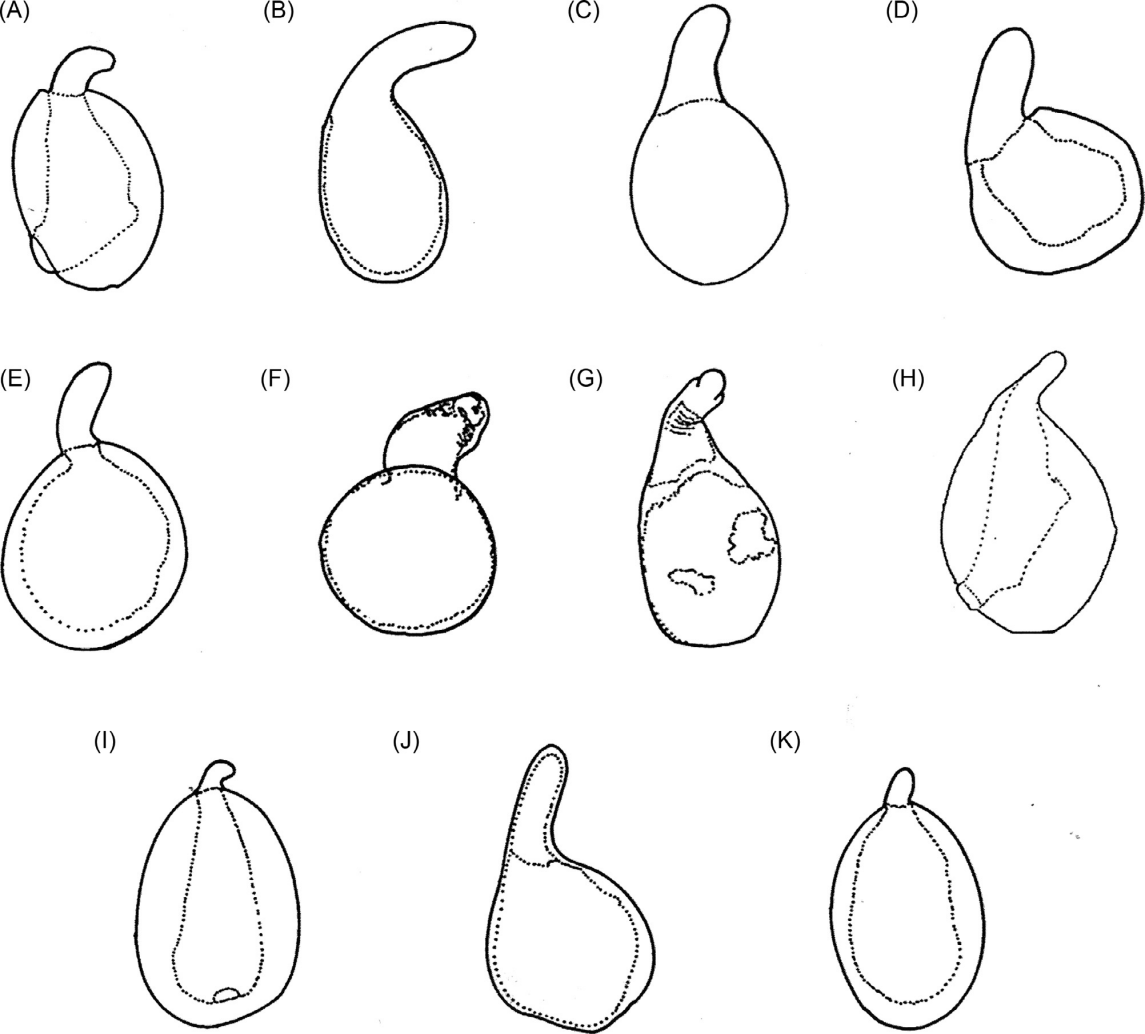
The spermathecae of some species of *Tunga*: (A) *T. penetrans*; (B) *T. caecata*; (C) *T. travassosi*; (D) *T. bondari*; (E) *T. terasma*; (F) *T. callida*; (G) *T. libis*; (H) *T. monositus*; (I) *T. trimamillata*; (J) *T. bossii*; (K) *T. hexalobulata*.

Figure 7 shows the spermathecae of 11 species. *T. travassosi*, *T. bondari*, *T. terasma*, *T. callida*, *T. monositus*, and *T. bossii* exhibit *bulgae* that are rounded and spherical, while in *T. penetrans*, *T. caecata*, *T. libis*, *T. monositus*, *T. trimamillata*, and *T. hexalobulata* they are elongated and ellipsoidal. Spermatecae with short hillae are seen in *penetrans*, *trimamillata*, and *hexalobulata*, while long hillae are found in *caecata*, *travassosi*, *bondari*, *terasma*, *callida*, *libis*, and *bossii*. On the other hand, the width of the hilla may be thin (*penetrans*, *terasma*, *monositus*, *trimamillata*, and *hexalobulata*) or thick (*bondari*, *callida*, and *libis*). The measurements of the bulga and hilla of the spermathecae are presented in Table 4.

**Table 4.** Morphological and morphometric data of modified segments of *Tunga* species.

*Tunga* species	Segment IX (clasper) of male			Female: spermatheca (µm) l* × w**
	Manubrium shape	Length manubrium (M)/basimere (B)/ratio	Width basimere (B)/telomere (T) ratio	
*T. penetrans*	Proximal portion wide; ventral margin straight and dorsal margin slightly convex.	M twice as long as B	B as wide as T	Bulga: 217.5 × 142.5; hilla: 62.5 × 32.5
*T. caecata*	–	–	–	Bulga: 240 × 197.5; hilla: 115 × 60
*T. travassosi*	–	–	–	Bulga: 200 × 161; hilla: 114 × 53
*T. terasma*	Proximal portion tapering and facing up; ventral margin concave and dorsal margin convex.	M twice as long as B	B as wide as T	Bulga: 190 × 150; hilla: 90 × 40
*T. bondari*	–	–	–	Bulga: 210 × 225; hilla: 160 × 65
*T. caecigena*	Proximal portion wide; ventral margin slightly concave and dorsal margin slightly convex.	M as long as B	B wider than T	–
*T. callida*	Proximal portion tapering; dorsal and ventral margins straight.	M twice as long as B	B wider than T	–
*T. libis*	Proximal portion blunt and tapering uniformly to a rounded apex; ventral margin concave and dorsal margin straight.	M as long as B	B as wide as T	–
*T. monositus*	Proximal portion truncate and facing down; ventral margin concave and dorsal margin convex.	M a little longer than B	B less wide than T	Bulga: 225 × 180; hilla: 55 × –
*T. trimamillata*	Proximal portion tapering and facing up; ventral margin straight and dorsal margin slightly convex	M twice as long as B	B as wide as T	Bulga: 295.2 × 201.7; hilla: 59 × 22.1
*T. bossii*	–	–	–	Bulga: 61.5 × 66.4; hilla: 41.8 × 12.3
*T. bonneti*	Proximal portion acuminate; dorsal margin straight and ventral margin concave.	M as long as B	B less wide than T	Bulga: 230 × ; hilla: 132 × –
*T. hexalobulata*	–	–	–	Bulga: 310.2 × 225; hilla: 54.3 × 35.3

l* = length; w** = width.

**Figure 8.**
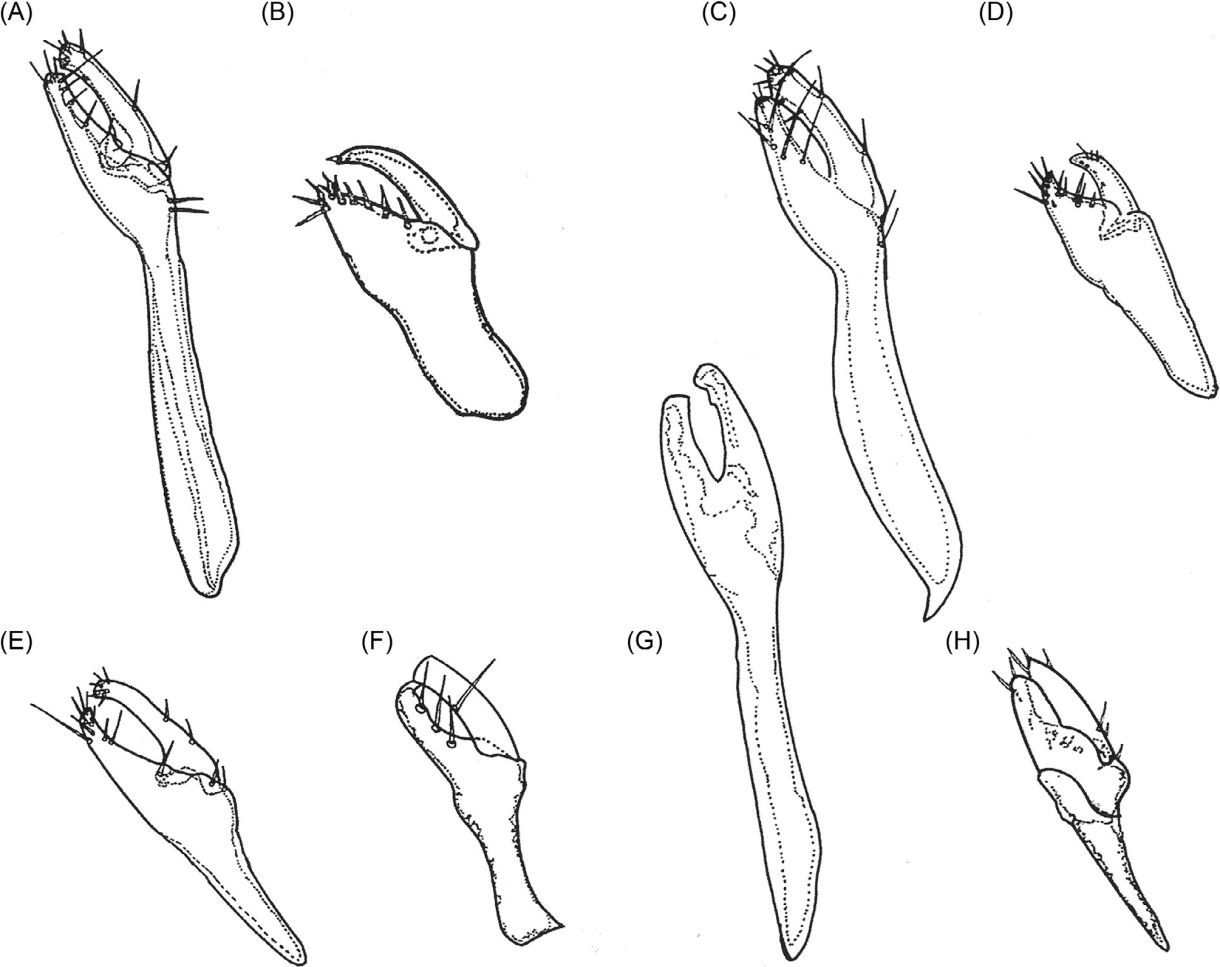
Segment IX of the males of some species of *Tunga*: (A) *T. penetrans*; (B) *T. caecigena*; (C) *T. terasma*; (D) *T. callida*; (E) *T. libis*; (F) *T. monositus*; (G) *T. trimamillata*; (H) *T. bonneti*.

In males, *segment IX (clasper)* and the *phallosome* are the most striking features for differentiating species. Similar to other fleas, *segment IX* is divided into two processes, the *basimere* (also often called the immovable or fixed process, or even p^1^) and the *telomere* (also called the movable process, movable finger, or even p^2^) which are connected to another structure, the *manubrium*. Figure 8 shows the shape and chaetotaxy of segment IX for the eight species whose males are known. The ratios between the width of the basimere and telomere in its median portion and the length of basimere/manubrium, and the shape of the basal and dorsal part of the manubrium are observed in Table 4. The *phallosome* consists of proximal and distal arms articulated in the middle, just before the *ductus ejaculatorius* and is almost as long as the *penis-plate*.

**Figure 9.**
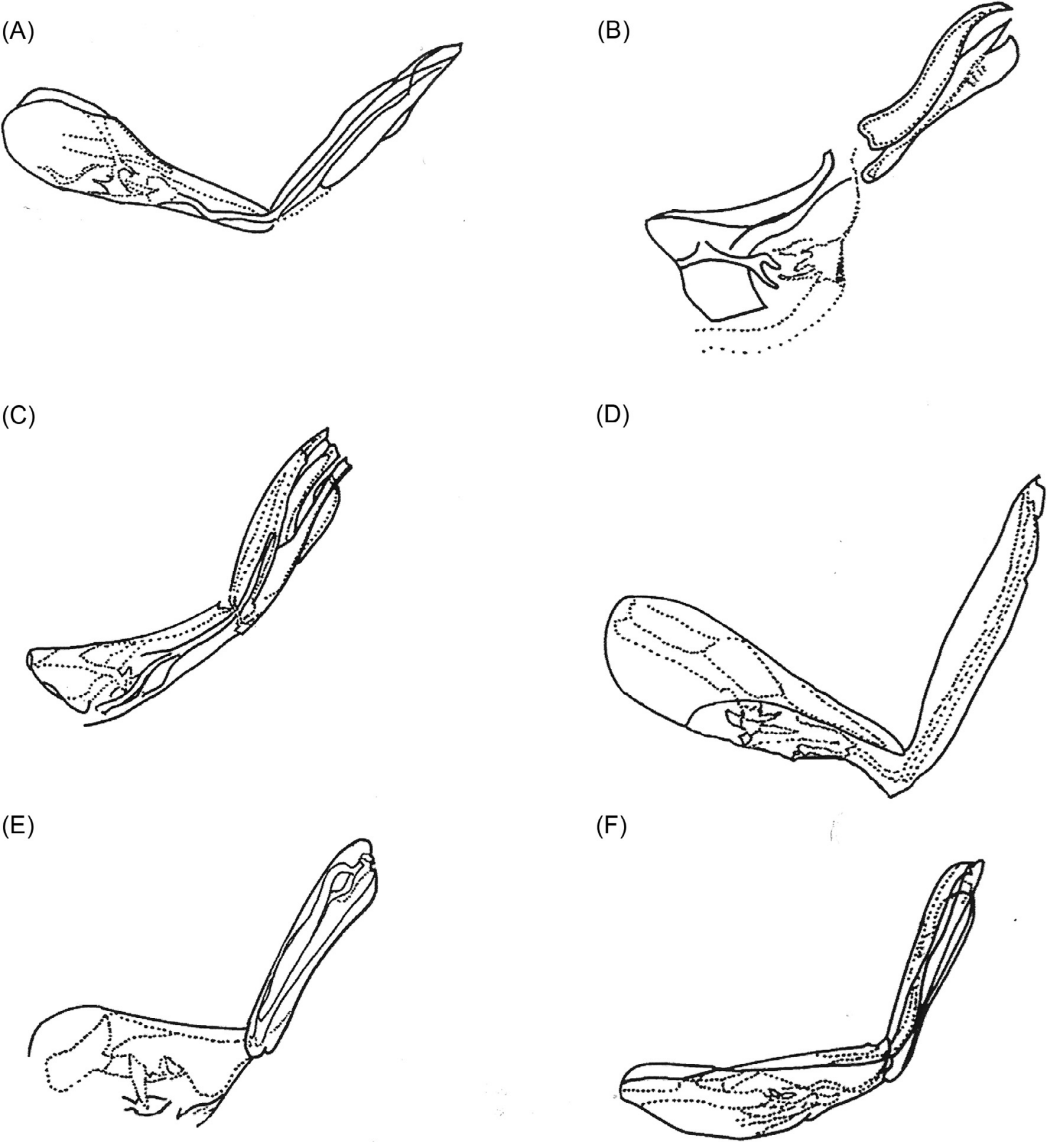
Male phallosomes of some species of *Tunga*: (A) *T. penetrans*; (B) *T. caecigena*; (C) *T. callida*; (D) *T. monositus*; (E) *T. trimamillata*; (F) *T. bonneti*.

The size ratio and the angle between the two arms are differential features for the species, as seen in Figure 9. In *T. penetrans* and *T. monositus*, the two arms are angled at approximately 90°, whereas in *T. callida* and *T. caecigena*, they are connected in a nearly straight line. Table 4 exhibits some morphologic features and morphometric data of the modified segments of the male and the spermathecae of the females.

## Taxonomy

The principal synonymies, sexes, individual variations, undescribed species, subgenera, higher taxa and a key for adult species and neosomes are included and discussed below.

### Synonymies

It is interesting to note that the older the description of the species, the greater the number of existing synonymies. Thus, *T. penetrans* presents seven major synonyms followed by *T. caecata* with two of them. All the synonymies for the genus were already cited by Beaucournu et al. [5].

### Sexes

As shown in Table 1, the species *caecata*, *travassosi*, *bondari*, *bossii*, and *hexalobulata* are known only through their hypertrophied females. *T. penetrans*, *T. callida*, *T. monositus*, *T. trimamillata*, and *T. bonneti* are species in which the holotype and the allotype were described simultaneously. In *caecigena* and *libis*, the allotype males were described 37 (1958) and 6 (1968) years, respectively, after the holotype females. The males of *T. terasma* were described incorrectly by Fonseca [17] as males of *T. travassosi*. Interestingly, the holotype female of *T. terasma* was described the following year by Jordan. It is possible that this is the only case in the entire order of Siphonaptera in which the allotype was known before the holotype.

### Individual variations

Regardless of sexual dimorphism (length, size of spiracles, chaetotaxy, maxillary palps), most of the individual variations are found in the modified segments, especially among the males. Hopkins and Rothschild [20] illustrate variations in the manubrium of *T. penetrans* and *T. terasma*. The illustrations of the male of *T. caecigena* presented by Chen and Ku [9] also show variations in the shape of the manubrium, basimere, and telomere. The figures of Wang [51] stress variations in the form of denticles on the distal portion of the basimere of *T. caecigena*. Similarly, when describing *T. monositus*, Barnes and Radovsky [2] illustrated and drew attention to the fact that the manubrium presents a highly variable shape, even on two sides of the same individual, with the proximal portion ranging from broad and blunt to slender, curved, and acuminate.

### Undescribed species

1. As reported in Linardi and Guimarães [34], hypertrophied females of the *caecata* group were observed by Linardi and Botelho [31] to parasitize *Oryzomys nigripes* (currently *Oligoryzomys nigripes*) and *Nectomys squamipes* from Caratinga, Minas Gerais State, Brazil, with the neosomes located near the base of the dorsal surface of the ears. The neosomes of *O. nigripes* are spherical and 8–9 mm in diameter, in contrast to those observed in *N. squamipes* that present three anterior humps and measure 8 mm in diameter (Figs. 1A, B). For these authors, the neosomes were two undescribed new species, despite the form found on *O. nigripes* having been confused with *T. monositus*. At that time, *T. trimamillata,* with its three anterior humps in the neosome had not yet been described – that was to happen in 2002.
2. Beaucournu et al. [5] commented that *T. bossii* had been seen, but not described, in the province of Minas Gerais, by Reinhardt in 1853 (*teste* Burmeister, 1854, quoted and commented by Smit [46], who wrote “Mesomys spinosus (taxon not known to us) *suffers from sand-fleas which preferably burrow near the anus and genitalia, at the base of the tail. This is very reminiscent of the habit of the recently described Chinese* Tunga callida *and I wonder whether indeed similar species do occur in Brazil*”. In the same paragraph, Burmeister informs us that Dr. Reinhardt “*Zeigte mir in Lagoa Santa (Minas Gerais) eine Hausmaus* (*=* Mus musculus), *die 13 solcher Flöhe an einem und 12 am anderen Ohr hatte*” (*He showed me a house mouse* (Mus musculus) *in Lagoa Santa (Minas Gerais, Brazil) that had 13 of these fleas on one ear and 12 on the other*). Burmeister and Reinhardt apparently caught sight of the two *Tunga* parasites of rodents in Brazil, *caecata* and *bossii*!
3. When defining neosomy, Audy et al. [1] included the figure of a neosome of an undescribed species that is morphologically similar to the neosome of *T. bonneti*. Subsequently, Frank Radovsky, one of the authors of this work (*in litt*., November 13, 1979 addressed to P.M. Linardi), reported that “*Incidentally, I have preserved material of* Tunga *in Phyllotis from Peru which I hope to describe. There are 2 undescribed species, one on the ear pinna as in* T. monositus *and your* Tunga *and one in the tail!*” However, these two species were ultimately not described by Radovsky. Given the morphological similarities, the hosts, and geographical distribution, it is possible that these two species were, in reality, *T. libis* and *T. bonneti*!
4. A new species of *Tunga* and belonging to the “*penetrans* group” was found in Argentina [16]. Subsequently Marcela Lareschi (*e-mail*, May 30, 2014 addressed to P.M. Linardi) reported that this species is now being described and that it forms a discoid neosome in the carapace of *Zaedyus pichiy* perforating the osteoderms.

### Subgenera

When Smit [46] divided the genus *Tunga* into two groups of species, he considered morphological characteristics such as the dorsal fusion of the pronotum and the mesonotum, the chaetotaxy of the fifth tarsal segment and the presence or the size of spiracular fossae on terga II–IV of the females, in addition to the parasitized host groups. At that time, only eight species were known. Four were included in the *penetrans* group (*T. penetrans*, *T. travassosi*, *T. bondari*, and *T. terasma*) and four others in the *caecata* group (*T. caecata*, *T. caecigena*, *T. callida*, and *T. libis*). Later, another proposal by Wang [51], based solely on geographical distribution, included the two known Chinese species, *caecigena* and *callida,* in a distinct subgenus (*Brevidigita*). When presenting the supraspecific classification for the genus *Tunga*, Lewis [28] accepted Wang’s proposal, but seems to have taken into consideration only those *Tunga* species that parasitize commensal rats for inclusion in *Brevidigita* because only *T. caecata* was added, though two other species, *T. monositus* and *T. libis*, were already known and were improperly left in the subgenus *Tunga*. Currently, with 13 known species, we consider the genus divided into two subgenera, *Tunga* and *Brevidigita*, though this division may be debatable:

Subgenus *Tunga*: *T. (T.) penetrans*, *T. (T.) travassosi*, *T. (T.) bondari*, *T. (T.) terasma*, *T. (T.) trimamillata*, and *T. (T.) hexalobulata*.
Subgenus *Brevidigita*: *T. (B.) caecata*, *T. (B.) caecigena*, *T. (B.) callida*, *T. (B.) libis*, *T. (B.) monositus*, *T. (B.) bossii*, and *T. (B.) bonneti*.

### Higher taxa

The vast majority of the Siphonaptera show a certain consistency for certain wildcard characteristics; for example, the profile of the cephalic capsule, development of the eye (unless a host to a different ecology), the proportions of the various articles of the palp, or the form of the spermatheca. However, in the genus *Tunga*, these rules are not respected. As has been done in the genus *Pulex* (*Pulex, Juxtapulex*…), further divisions may be required when the puzzle of taxa known as *Tunga* is complemented by a comparative study of all males.

Beaucournu (*in* Aberlenc) [3]) recognizes Siphonaptera as

“*insect holometabolous, wingless larvae apoda detritiphages but with mandatory addition of blood in their diet in adulthood latero-lateral, flattened to hind legs longer than the 2 prior to biting mouthparts (hematophagous regime), size ranging, fasting and/or before fertilization, from 0.75 to 8 mm.* Now, we are also including “*bristles directed backward and brownish colour*”.

*- During copulation, the male is always under the female. The phallosome has no middle joint, except in* Hectopsylla. *The female is vagile, more rarely sessile, and never cystic in the dermis of the host* (*except in* Neotunga euloidea [*Pulicidae*] *in which the male is not known*); *the female’s spermatheca is completely visible before any meal or copulation; the male’s abdomen may become neosomic, but it will always remain outside the host* (*except in N*. euloidea); *the abdominal spiracles do not change in shape, size or location during the life of the imago even in the case of neosomy*… ***Pulicoidea, Vermipsylloidea, Malacopsylloidea, Ceratophylloidea and Hystrichopsylloidea***

*- During copulation, the male is always on the female. The phallosome presents a middle joint. The female is vagile only while searching for a host, and upon finding one it becomes completely encapsulated; the female’s spermatheca is visible in its entirety, only when encapsulated* (bulga *and* hilla); *female neosomy is very important and compulsory; abdominal stigma in the neosomes, are very enlarged and displaced and form on the terminal tergites*… ***Tungoidea***

For this author, the Tungoidea comprises only the genus *Tunga*. *Hectopsylla* remains in the Pulicoidea. Linardi asserts (as seen in Linardi and Guimarães [34]) that *Hectopsylla* must be retained in Tungidae, as evidenced by DNA analysis [52].

### Key for adults and neosomes

The first dichotomous key for this genus was that of Hopkins and Rothschild [20] and did not include six species that were subsequently described (c*allida*, *libis*, *monositus*, *trimamillata, bossii, bonneti*, *and hexalobulata*). While it is no longer usable, it proposed to differentiate Tungidae, but using characteristics that have proved worthless, for example, the indentation of the eye. This same key, excluding the two Chinese *Tunga*, was later reproduced by Johnson [21] to identify South American sand fleas. Barnes and Radovsky [2], when describing *T. monositus*, presented a key exclusively for the *caecata* group species known at that time.

Recently, De Avelar et al. [13], when describing *Tunga bossii*, proposed a new key, including all known taxa (both *bonneti* and *hexalobulata* are absent since their descriptions appeared after *bossii*) in which the appearance of the neosome is widely used. Although often the morphological aspects of many taxa that we would like to use in classification are not known (the autotomy of legs is mainly in females), we attempt to provide a more classical vision of differentiation, with emphasis on independent characteristics of neosomes. Thus, this new key also includes the means to differentiate known male characteristics.

1. Pronotum and mesonotum fused dorsally. Fifth tarsomere of the metatarsus with chaetotaxy reduced and exhibiting only two pairs of lateral plantar bristles (Fig. 6A). Males have a manubrium approximately two times longer than the basimere and exhibit a small constriction between them (Figs. 8F–H) (Subgenus ***Tunga***) ..............................................................................2
2. Pronotum and mesonotum not completely fused dorsally. Fifth tarsomere of the metatarsus with chaetotaxy not reduced and exhibiting more than three pairs of lateral plantar bristles (Figs. 6B–F). Males have a manubrium a little longer or as long as the basimere and without constriction between them (Figs. 8A–E) (Subgenus ***Brevidigita***)........................................................................7
3. Eye without black pigment (Fig. 3D). Base of maxilla with only a micro-bristle. Maxillary palp as in Figure 4D. Metacoxa as in Figure 5D. Spermatheca as in Figure 7C. Globular neosome with head invaginated within abdomen, measuring (mm) 12–13 (length), 8 (width), and 10 (height). Male unknown......................................................***T. (T.) travassos*** *i* Pinto and Dreyfus.
4. Eye pigmented (Figs. A, E, F, J, M). Base of maxilla with two or more bristles. Neosome with head not invaginated within abdomen or laterally visible........................................................................3
5. Eye small, its greatest diameter only slightly exceeding half the distance from eye to dorsal margin of head (Fig. 3E). Dorsal genal lobe covering part of antennal segment III. Segment IV of maxillary palp longer than segment I (Fig. 4E). Metacoxa as in Figure 5E. Spermatheca with bulga wider than long (Fig. 7D). Mushroom-shaped neosome with a stem and conical posterior region and measuring (mm) 6 (length), 6 (width), and 5 (height). Males unknown ......................................................................................................................***T. (T.) bondari*** Wagner.
6. Eye large, its greatest diameter nearly equal to the distance from eye to dorsal margin of head. Dorsal genal lobe not covering the antennal segment III. Segment I of maxillary palp longer than segment IV. Spermatheca with a bulga longer than wide. Neosome globular or with prominent lobes.................................................................................................................................................4
7. Frontal tubercle slightly pronounced (Fig. 3F). Antennal segment II with only two bristles. Maxillary palp with few bristles (Fig. 4F). Metacoxa with proximal portion as wide as distal portion (Fig. 5F). Manubrium with ventral margin concave and dorsal margin convex (Fig. 8C). Spermatheca with globular bulga two times longer than hilla (Fig. 7D). Hypertrophied female not globular and containing four prominent lobes, measuring (mm) 10 (length), 9 (width), and 13 (height) and with cylindrical caudal disk, longer than wide................................................................................................................***T. (T.) terasma*** Jordan.
8. Frontal tubercle conspicuous (Figs. 3A, J, M). Antennal segment II with more than two bristles. Maxillary palp with numerous bristles (Figs. 3A, J, M). Metacoxa with proximal portion wider than distal portion (Figs. 5A, H, M). Manubrium with ventral margin straight and dorsal margin a little convex (Figs. 8A, G). Spermatheca with ellipsoidal bulga and more than three times longer than hilla. Female is globular and hypertrophied or exhibits three or six anterior lobes, with flattened or conical caudal disk, wider than long............................................................................5
9. Head with pronounced ventral genal lobe (Fig. 3A). Anterior base of maxilla with three thick bristles. Segment II of maxillary palp longer than segment I (Fig. 4A). Anteromedial surface of posterior tibia without bristles. Manubrium with proximal portion wide (Fig. 8A). Phallosome as Figure 9A. Bulga of spermatheca approximately 3.5 times longer than hilla (Fig. 7A). Globular hypertrophied female without projections or lobes and with flattened caudal disk, measuring (mm) 6 (length), 5 (width), and 5 (height)..................***T. (T.) penetrans*** (L.).
10. Head with no evidence of ventral genal lobe (Figs. 3J, M). Anterior base of maxilla with 6–7 bristles. Segment I of maxillary palp longer than segment II (Figs. 4I, L). Bulga of spermatheca almost six times longer than hilla (Figs. 7I, K). Globular hypertrophied female with three or six anterior lobes and conical caudal disk .........................................................................................................6
11. Head with the postantennal region exhibiting 17–19 bristles (Fig. 3I). Antennal segment II with four bristles. Segment IV of maxillary palp almost as long as segment II (Fig. 4I). Metacoxa without a projection at the margin of the proximal portion (Fig. 5H). Spermatheca as in Figure 7I, with curved hila and bulga nine times wider than hilla. Anteromedial surface of posterior tibia with row of 9–12 bristles. Manubrium with proximal portion tapering and facing up (Fig. 8G). Phallosome as Figure 9E. Neosome with three lobes located anteriorly, measuring (mm) 12 (length), 5 (width), and 5 (height)...............................................................................................................***T. (T.) trimamillata*** Pampiglione, Trentini, Fioravanti, Onore, and Rivasi.
12. Head with the postantennal region exhibiting nine bristles (Fig. 3M). Antennal segment II with three bristles. Metacoxa with a projection at the margin of the proximal portion (Fig. 5K). Spermatheca as Figure 7K, with straight hilla and bulga six times wider than hilla. Males unknown. Neosome with six lobes located anteriorly, measuring (mm) 4 (length), 4 (width), and 4 (height).................................................*T. (T.) hexalobulata* De Avelar, Facury Filho, and Linardi.
13. Eye absent. Males with the basal and apical tubes of the phallosome articulated almost in a straight line (Figs. 9B–C).................................................................................................................8
14. Eye present. Males with the basal and apical tubes of the phallosome forming a right angle (Figs. 9D, F)...............................................................................................................................................9
15. Occipital region with 8–10 bristles (Fig. 3C). Preoral internal sclerotization with anterior arm extending to half the distance between frontal tubercle and base of maxillary palp. Lacinia as long as maxillary palp. Manubrium almost as long as basimere (Fig. 8B). Phallosome as in Figure 9B. Elliptical neosome, with dorsal and ventral portions of similar dilatation, measuring (mm) 7–10 (length), 5 (width), and 6 (height) and caudal disk longer than wide...................................................................................***T. (B.) caecigena*** Jordan and Rothschild.
16. Occipital region with 3–5 bristles (Fig. 3G). Preoral internal sclerotization with anterior arm extending near the base of maxillary palp. Lacinia longer than maxillary palp. Manubrium slightly longer than basimere (Fig. 8D). Phallosome as in Figure 9C. Spherical neosome, with the dorsal portion more swelled than the ventral portion, measuring (mm) 4.5 (length), 4.5 (width), and 4.5 (height) and caudal disk as long as wide.......................***T. (B.) callida*** Li and Chin.
17. Eye pigmented. Head with the frontal curvature almost straight (Figs. 3I, L). Manubrium with the proximal portion tapering, acuminate (Figs. 8E, H). Elliptical gravid female........................10
18. Eye without black pigment. Head with the frontal curvature gently convex (Figs. 3B, I, K). Manubrium with the proximal portion truncate. Globular or bell-shaped gravid female..............11
19. Fifth metatarsomere with numerous minute plantar bristles (Fig. 6D). Segment II of the maxillary palp longer than segment III. Telomere as wide as basimere (Fig. 8E). Vertically elliptical neosome, higher than long and without lobes...........................................***T. (B.) libis*** Smit.
20. Fifth metatarsomere of the metatarsus with few minute plantar bristles (Fig. 6F). Segment II of the maxillary palp as long as segment III (Fig. 4K). Telomere wider than basimere (Fig. 8H). Horizontally elliptical neosome, longer than high, rugby-ball shaped and without lobes.....................................................................***T. (B.) bonneti*** Beaucournu and González-Acuña.
21. Posterodorsal lobe of proepimeron large, strongly projecting. Base of maxilla with no bristles. Metacoxa with basal portion much wider than apical and with anterior basal corners dilated (Fig. 5G). Spermatheca with bulga four times longer than hilla (Fig. 7H). Males with segment IX as in Figure 8F and phallosome as in Figure 9D. Bell-shaped gravid female with eight lobes, arranged as four large outer lobes and four small inner lobes......................................................................................................................***T. (B.) monositus*** Barnes and Radovsky.
22. Posterodorsal lobe of proepimeron small, rounded. Base of maxilla with one or two bristles. Metacoxa with basal portion a little wider than apical and without dilatation in the anterior basal corners (Figs. 5B, I). Spermatheca with bulga that is two times longer than hilla (Figs. 7B, J). Males unknown. Globular gravid female without lobes................................................................12
23. Preoral internal sclerotization with posterior arm more than eight times the length of anterior arm (Fig. 3B). Frontal tubercle slightly pronounced. Eye with the greatest diameter less than half the length of the antennal segment II. Base of maxilla with one bristle. Segment IV of the maxillary palp less than the length of segments II + III (Fig. 4B). Metacoxa as in Figure 5B. Spermatheca with bulga longer than wide and hilla two times longer than wide (Fig. 7B). Neosome, measuring (mm) 7 (length), 6 (width), and 6 (height) and conical caudal disk, almost as wide as long.........................................................................................................***T. (B.) caecata*** (Enderlein).
24. Preoral internal sclerotization with posterior arm less than four times the length of anterior arm (Fig. 3K). Conspicuous frontal tubercle. Eye with the greatest diameter almost as long as antennal segment II. Base of maxilla has two bristles. Segments II, III, and IV of the maxillary palp undivided, but apparently IV greater than II + III (Fig. 4J). Metacoxa as in Figure 5I. Spermatheca with bulga wider than long and hilla about four times longer than wide (Fig. 7J). Neosome, measuring (mm) 9 (length), 8 (width), and 7 (height) and flattened caudal disk, wider than long......................................................***T. (B.) bossii*** De Avelar, Linhares, and Linardi.

## Final remarks

The geographical center of the distribution of the genus *Tunga* is concentrated in South America, including 77% of the species that most likely originated there. Because approximately 23% of the species were described in the last 2 years from Ecuador, Brazil, and Chile, the biomes and regions contained in these countries offer great opportunities for new findings. There are at least three or four undescribed species, with one of them awaiting description and another two or three remaining confused with species that are already known.

Thirty-nine percent of the species are known only by the characteristics of the embedded females (neosomes). Only two out of the 13 described species of sand fleas have known larvae, although several larvae of the 1st instar of an undescribed species were obtained in the laboratory by Linardi and Botelho [34] from hypertrophied females infesting Brazilian wild rodents. Consequently, investigation of the alternate sexes and immature forms provides promising lines of research in the respective regions of their occurrences.

Because larval development occurs in fine-grained soils and the sand fleas are univoltine, the best results can be obtained in the dry-cool season.

Given the epidemiological and economic importance of *T. penetrans* and *T. trimamillata,* which infest both domestic animals and humans, the correct identification of species is indispensable. *T. penetrans* has been found parasitizing at least 28 genera of hosts [10], although some occurrences are incorrect records [32, 33]. Morphological variations must be used carefully for taxonomic purposes. Molecular biology should also be used for such purposes. As showed by De Avelar and Linardi [12] the Multiple Displacement Amplification technique (MDA) may be a valuable tool for molecular studies involving samples of sand fleas that are preserved in scientific collections.

Rodents and edentates are the main hosts of Siphonaptera, housing approximately 85% of the known species.

Data on host specificity and geographical distribution may support the identification of *Tunga* species because some sand fleas and their hosts may have co-evolved. According to Traub [48], most fleas have clearly evolved with their hosts and primitive hosts tend to have primitive fleas [47]. Mammals such as Edentata might have been the primitive hosts of tungids because they are devoid of incisor teeth and nails to remove the neosomes attached on toes and on the ventral abdominal, regions regularly in contact with the soil and of great difficulty for the hosts to dislodge the parasites by grooming or eating. On the other hand, based on the molecular phylogeny, Whiting et al. [52] placed *Tunga* at the base of flea phylogeny and its association with basal mammal hosts suggests that the origin and diversification of Siphonaptera coincided with basal mammal diversification. Sloths (Pilosa) and armadillos (Cingulata) belong to an ancient stock of mammalians and constitute the majority of the natural mammalian hosts of the genus *Tunga* [52].

## Conflict of Interest

No conflict of interest.
